# The metastasis promoting protein S100A4 levels associate with disease activity rather than cancer development in patients with idiopathic inflammatory myopathies

**DOI:** 10.1186/s13075-014-0468-2

**Published:** 2014-10-31

**Authors:** Lenka Pleštilová, Heřman Mann, Lucie Andrés Cerezo, Ondřej Pecha, Jiří Vencovský, Ladislav Šenolt

**Affiliations:** Institute of Rheumatology, 12850 Prague, Czech Republic; Department of Rheumatology, First Faculty of Medicine, Charles University in Prague, Prague, Czech Republic; Technology Centre of the Academy of Sciences, Prague, Czech Republic

## Abstract

**Introduction:**

The aim was to evaluate S100A4 protein as a biomarker of disease activity and potential cancer development in patients with myositis.

**Methods:**

Serum levels of S100A4 were determined in 43 dermatomyositis (DM), 39 polymyositis (PM) and 22 cancer associated myositis (CAM) patients as well as in 77 healthy controls. The associations between S100A4 levels, inflammation, disease activity, muscle strength and cancer development were evaluated.

**Results:**

All myositis patients had significantly higher serum levels of S100A4 protein compared to healthy controls (median (IQR): 31.5 (17.4 to 59.5) versus 23.8 (14.5 to 33.7) ng/ml, *P* <0.05). In patients with PM, serum levels of S100A4 protein were significantly higher than in healthy controls (41.6 (24.2 to 123.1) versus 23.8 (14.5 to 33.7) ng/ml; *P* <0.001) as well as in patients with DM (26.7 (11.3 to 47.5) ng/ml; *P* <0.05). The levels of S100A4 were comparable between myositis with and without cancer. In all myositis patients, serum S100A4 levels correlated with MYOsitis disease ACTivity assessment (MYOACT) score (r = 0.34; *P* = 0.001), constitutional (r = 0.30; *P* = 0.003), pulmonary (r = 0.43; *P* = 0.0001) and extramuscular disease activity (r = 0.36; *P* = 0.0001), as well as with creatine phosphokinase (r = 0.27; *P* = 0.015) and lactate dehydrogenase (r = 0.37; *P* = 0.002) or c-reactive protein (CRP) levels (r = 0.24; *P* = 0.038). Multiple regression analysis showed significant association between S100A4 serum levels and extramuscular disease activity (β = 0.552; *P* = 0.002) in PM patients and with MYOACT (β = 0.557; *P* = 0.003) and CRP levels (β = 0.391; *P* = 0.029) in DM patients.

**Conclusions:**

Circulating levels of S100A4 are elevated in patients with myositis and associate with several disease activity parameters, particularly with extramuscular components. No relation between S100A4 levels and presence of cancer associated myositis was found.

**Electronic supplementary material:**

The online version of this article (doi:10.1186/s13075-014-0468-2) contains supplementary material, which is available to authorized users.

## Introduction

Idiopathic inflammatory myopathy is a heterogeneous group of chronic muscle disorders with main subtypes including polymyositis (PM), dermatomyositis (DM), inclusion body myositis and necrotizing myopathy [1]. The diagnosis of myositis is based on the combination of symmetrical muscle weakness accompanied by elevation of circulating muscle enzymes, characteristic electromyography (EMG) and muscle biopsy findings. Extramuscular manifestations of myositis are common and include interstitial lung disease, dysphagia or arthritis, and the presence of distinctive skin rash in patients with DM [2]. An association of idiopathic inflammatory myopathy with malignancy has been documented in a number of studies [3-5]. However, the molecular link explaining the increased risk of cancer in myositis patients is still poorly understood [6,7].

S100A4 protein represents an important member of the S100 family of small calcium-binding proteins [8,9]. Interacting with several target proteins, S100A4 affects a number of activities, accelerating tumorigenesis and invasion of human cancers. At the molecular and cellular level, the cancer-promoting properties of S100A4 are caused by regulating cell motility, proliferation, apoptosis, and by stimulation of angiogenesis and remodelling of the extracellular matrix [10-14]. The expression of S100A4 protein correlates with the patient’s prognosis in breast cancer [15], colorectal cancer [16] and variety of other tumors [10,11]. We and others have recently demonstrated increased expression of S100A4 at local sites of inflammation in several chronic inflammatory and autoimmune diseases [17-21], including muscle tissue from patients with idiopathic inflammatory myopathies [22]. Our results showed, that in inflamed muscle, the S100A4 protein is produced mainly by mononuclear cells present in the inflammatory infiltrates, by endothelial cells and by regenerating muscle fibres [22]. Moreover, we have previously found increased circulating levels of S100A4 in patients with rheumatoid arthritis (RA) in comparison with control individuals and demonstrated a positive correlation between S100A4 and disease activity in RA [23].

Based on these findings we have conducted a study in order to determine the S100A4 serum levels in myositis patients, to evaluate the association between circulating S100A4 and myositis disease activity and to assess a potential role of S100A4 in cancer-associated myositis (CAM).

## Methods

### Patients and disease activity assessment

A total of 104 patients with myositis (43 with DM, 39 with PM and 22 with CAM) and 77 healthy controls were enrolled in the study. Longitudinal serum samples were available for 11 patients. The interval between the two blood withdrawals was 9 ± 6 months. Myositis patients were recruited from a single centre of the inpatient and outpatient departments of the Institute of Rheumatology in Prague. The diagnosis of DM and PM was based on the Bohan and Peter criteria [24,25]. CAM was defined as cancer occurring within 3 years of the diagnosis of myositis. All individuals gave informed consent to participate and the study was approved by the Ethics Committee of the Institute of Rheumatology in Prague.

Clinical disease activity was evaluated by the disease activity core set measures proposed by International Myositis Assessment & Clinical Studies Group (IMACS): myositis disease activity assessment (MYOACT) and physician global activity using visual analogue scales (VAS), manual muscle testing (MMT) and the health assessment questionnaire (HAQ) [26]. Muscle biopsies performed within one month from the blood withdrawal were available for 13 patients with PM.

### Laboratory measurements

Blood samples were collected from all patients and control individuals and stored at −80°C until analysis. Serum S100A4 concentrations were measured by an ELISA kit according to the manufacturer’s protocol (CycLex Co, Ltd, Ina, Nagano, Japan) as demonstrated elsewhere [23]. The analysis was performed using the ELISA reader SUNRISE (Tecan, Salzburg, Austria) at a wavelength of 450 nm.

Serum levels of the muscle-associated enzymes creatine phosphokinase (CK) and lactate dehydrogenase (LD), as well as C-reactive protein (CRP) were measured by routine laboratory techniques. Myositis specific and associated autoantibodies were detected by immunoprecipitation as described elsewhere [27].

### Statistical analyses

The data were described as median (IQR) if variables were not normally distributed and as mean (SD) if normally distributed. The Kruskal-Wallis test and corresponding post-hoc analysis and Mann-Whitney test were conducted for comparison between groups. Before analysis of associations among variables, the data were first transformed toward normality. Logarithmic transformation was used because all variables were positively skewed. Pearson’s product-moment correlation coefficients were calculated to quantify relationships between disease activity, laboratory markers and S100A4 levels. All correlations were adjusted for age, body mass index, and disease duration using the partial correlation technique. Multiple linear regression analysis was performed to establish whether specific factors were simultaneously associated with the S100A4 as a dependent variable. Covariates included age, sex and disease duration, as well as laboratory markers (CRP, CK and LD) and disease activity measures (MYOACT and its components, extramuscular and muscle disease activity, physician global disease assessment, HAQ and MMT8) were considered as independent variables. The appropriate predictors were chosen using a backward stepwise elimination method. Missing data were excluded using pairwise deletion. *P*-values <0.05 were considered statistically significant. A correlation coefficient of 0.1 to 0.3 was considered weak, 0.3 to 0.5 was considered moderate and 0.5 to 1.0 was considered strong correlation. The analysis was performed using SPSS 17.0 (SPSS, Chicago, IL, USA) and the graphs were prepared using GraphPad Prism (version 5.00 for Windows, GraphPad Software, San Diego, CA USA, [28]).

## Results

### Characteristics of patients

The characteristics of myositis patients and healthy controls are summarized in Table 1, and types of cancer in patients with CAM are given in Table 2. A majority of patients with CAM had DM (n = 18). Disease duration ranged from 0 to18 months from diagnosis. Some patients were treated for specific overlap syndromes, including myositis overlap with systemic lupus erythematosus (n = 3) and systemic sclerosis (n = 3). Two patients with immune-mediated necrotizing myopathy (IMNM) were included in the PM subgroup, as they fulfilled the Bohan and Peter classification criteria [24,25]. All patients had proximal muscle weakness. Rash was present in 57%, mechanics hands in 35% and Raynaud’s phenomenon in 21% of patients. Arthritis was diagnosed in 31% and dysphagia in 44% of patients. Myositis-associated interstitial lung disease and cardiac involvement was present in 40% and 18% of patients, respectively. The anti-TIF1 autoantibodies, known to be associated with malignancy, were detected in 17 myositis patients (5 with DM, 1 with PM and 11 with CAM) [29]. Fifteen patients were treatment-naive at the time of assessment. In the 84 patients treated with glucocorticoids at the time of blood withdrawal, the median dose was 15 mg of prednisone or its equivalent per day ranging from 2.5 to 100 mg per day. Twenty-seven patients received methotrexate in addition to glucocorticoids, six patients were treated with cyclosporine A, three with azathioprine and two with hydroxychloroquine (one in monotherapy). Four patients were not treated at the time of blood sample collection, but were on immunosuppressive drugs previously.

**Table 1:** **Characteristics and demographic data of patients with idiopathic inflammatory myopathies and healthy controls**

	**Dermatomyositis (DM)**	**Polymyositis (PM)**	**Cancer-associated myositis (CAM)**	**Healthy controls (HC)**
Number	43	39	22	77
Gender, female/male	35/8	25/14	20/2	58/19
Age, years	58 (41 to 63)	55 (36 to 60)	64 (59 to 70)	45 (31 to 54)
S100A4, ng/ml	26.7 (11.3 to 47.5)	41.6 (24.2 to 123.1)	28.8 (12.6 to 45.4)	23.8 (14.5 to 33.7)
S100A4, ng/ml in females	26.7 (9.0 to 54.7)	54.2 (25.4 to 115.6)	28.8 (13.0 to 47.5)	22.2 (13.6 to 32.1)
S100A4, ng/ml in males	30.3 (19.5 to 44.8)	41.3 (23.9 to 157.9)	20.3 (5.5 to 35.0)	32.3 (19.9 to 64.6)*
**Disease duration, years**	2.4 (0.4 to 8.3)	0.9 (0.5 to 3.2)	0.6 (0.2 to 3.6)	NA
**Biochemical markers**				
CRP, mg/L	3.3 (1.3 to 9.0)	4.7 (2.2 to 26.1)	8.0 (3.2 to 26.6)	NA
CK, ukat/L	2.6 (1.2 to 9.9)	11.6 (2.0 to 29.3)	10.9 (1.5 to 37.1)	NA
LD, ukat/L	4.4 (3.5 to 6.4)	7.1 (4.2 to 10.9)	5.2 (2.9 to 6.0)	NA
**Autoantibodies**	6*Mi-2	12*Jo-1;	11*TIF1	NA
	4*TIF1, 1*TIF1 + U1RNP	1* Jo-1 + U1RNP;	4*Mi-2	
	2*NXP2	1*Jo-1 + RNAPI + RNAPII	2*Jo-1, 1*Jo-1 + Ro	
	3*PM-Scl	2*PM-Scl	1*SAE	
	3*Jo-1	1*PL7	3*without known aAbs	
	19*without known aAbs	1*Ku		
		1*SRP		
		1*TIF1 + AMA		
		15*without known aAbs		
**Clinical features, number**				
Muscle weakness	43	39	22	NA
Rash	42	1	16	NA
Mechanic’s hands	18	10	8	NA
Raynaud’s phenomenon	9	10	3	NA
Arthritis	14	14	4	NA
Interstitial lung disease	15	22	5	NA
Cardiac involvement	10	6	3	NA
Dysphagia	21	15	10	NA
**Disease activity**				
MYOACT	0.6 (0.15 to 1.5)	0.7 (0.2 to 1.3)	0.8 (0.5 to 1.8)	NA
Constitutional DA, VAS	6 (0 to 18)	8 (0 to 24)	0 (0 to 28.5)	NA
Cutaneous DA, VAS)	15 (0 to 30)	0 (0 to 6)	28.5 (0 to 52.8)	NA
Skeletal DA, VAS	0 (0 to 0)	0 (0 to 7)	0 (0 to 2)	NA
Gastrointestinal DA, VAS	0 (0 to 4)	0 (0to3)	0 (0 to 25.5)	NA
Pulmonary DA, VAS	0 (0 to 7)	6.5 (0 to 24.7)	0 (0 to 13)	NA
Cardiac DA, VAS	0 (0 to 0)	0 (0 to 0)	0 (0 to 0)	NA
Extramuscular DA, VAS	15 (4 to 34)	17 (3.5 to 32.5)	20 (11.5 to 54.5)	NA
Muscle DA, VAS	10 (3 to 44)	32 (14 to 53)	37 (5.5 to 68.3)	NA
Physician’s global disease Assessment, VAS	19.5 (4.5 to 43.7)	30.5 (22.5 to 49.3)	42.5 (13.5 to 52)	NA
HAQ	1.0 (0.3 to 1.9)	0.9 (0.5 to 1.3)	1.0 (0.5 to 2.6)	NA
MMT8	62 (52 to 74)	71 (63 to 74.5)	56 (50.5 to 69.0)	NA
**Treatment**				
Treatment duration, months	13.6 (0.9 to 83.6)	2.0 (0.3 to 72.1)	1.4 (0.0 to 20.6)	NA
Daily glucocorticoid dosage, mg prednisone equivalent	10.0 (2.5 to 40.0)	25.0 (3.8 to 50.0)	6.3 (0.0 to 42.5)	NA
Immunosuppressive drugs	11*MTX, 3*Plaquenil, 2* AZA, 2*CyA, 23*no immunosuppressive drugs at the time of blood withdrawal, 2*before start of the immunosuppressive treatment	10*MTX,1*MTX + AZA, 1*MTX + SAS, 4*CyA, 2*Plaquenil, 1*AZA, 15*no immunosuppressive drugs at the time of blood withdrawal, 5*before start of the immunosuppressive treatment	4*MTX, 11*no immunosuppressive drugs at the time of blood withdrawal, 7*before start of the immunosuppressive treatment	NA

Data are presented as number or median (IQR). *S100A4 serum levels in males versus females, *P* = 0.025. NA, not applicable; CRP, C-reactive protein; CK, creatinine phosphokinase; LD, lactate dehydrogenase; aAbs, autoantibodies; MYOACT, myositis disease activity assessment; DA, disease activity; VAS, visual analogue scale; HAQ, health assessment questionnaire; MMT, manual muscle testing; MTX, methotrexate; AZA, azathioprine; CyA, cyclosporine A; SAS, sulfasalazine.

**Table 2:** **Types of cancer in patients with cancer-associated myositis**

**Type of cancer**	**Number of patients**
Breast cancer	8
Breast cancer + uterine cancer	1
Ovarian cancer	3
Tonsillar cancer	2
Uterine cancer	1
Prostate cancer	1
Hepatic cancer	1
Colorectal cancer	1
Sarcoma on the neck	1
Invasive thymoma	1
Metastatic cancer of unknown origin	1
Information missing	1

### Circulating S100A4 is elevated in myositis patients

**Figure 1:**
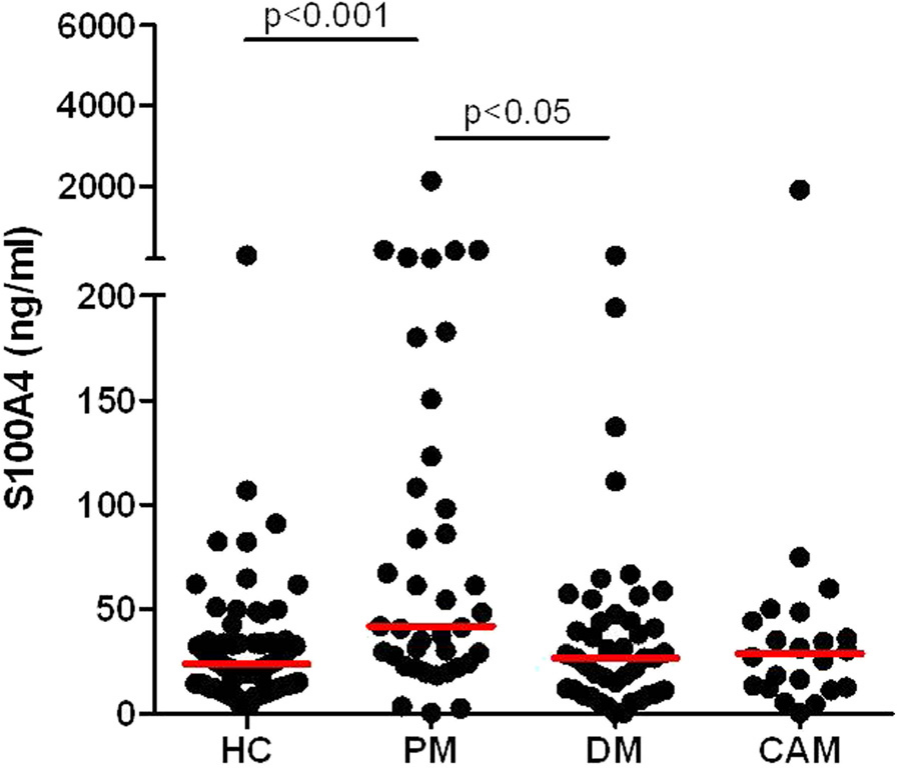
**Circulating S100A4 levels in patients with polymyositis (PM), dermatomyositis (DM), cancer-associated myositis (CAM) and healthy controls (HC).** Horizontal lines represent the median. The Mann-Whitney *U*-test was used to calculate *P*-values for differences between groups.

The levels of serum S100A4 were higher in all myositis patients than in healthy controls (31.5 (17.4 to 59.5) versus 23.8 (14.5 to 33.7) ng/ml, *P* <0.05). Patients with PM had higher serum levels of S100A4 compared to healthy controls (41.6 (24.2 to 123.1) versus 23.8 (14.5 to 33.7) ng/ml, *P* <0.001) or to patients with DM (to 26.7 (11.3 to 47.5) ng/ml, *P* <0.05). There was not a significant difference between patients with DM or CAM and healthy controls (26.7 (11.3 to 47.5) or 28.8 (12.6 to 45.4) versus 23.8 (14.5 to 33.7) ng/ml, both *P* <0.05) (Figure 1).

The levels of serum S100A4 were higher in healthy male compared to female subjects, but there were no significant sex-related differences in S100A4 levels in myositis patients (Table 1). A weak inverse correlation between S100A4 levels and disease duration was observed in all myositis patients (*r* = −0.27; *P* = 0.006). In this context, patients who were treated for longer than 6 months (n = 50) compared with those who were treated for a shorter period of time (n = 54) had lower levels of S100A4 (22.0 (9.6 to 43.4) versus 38.7 (27.2 to 65.2) ng/ml, *P* = 0.002). However, serum levels of S100A4 did not correlate with daily dose of prednisone and were not affected by age. In addition, in longitudinal serum samples S100A4 levels decreased during the treatment; however, due to the limited number of samples for investigation (n = 11), this change was not statistically significant (86.4 (40.0 to 218.4) versus 45.2 (28.9 to 139.9) ng/ml, *P* = 0.195).

### Association between S100A4 levels and myositis-specific and associated autoantibodies

**Figure 2:**
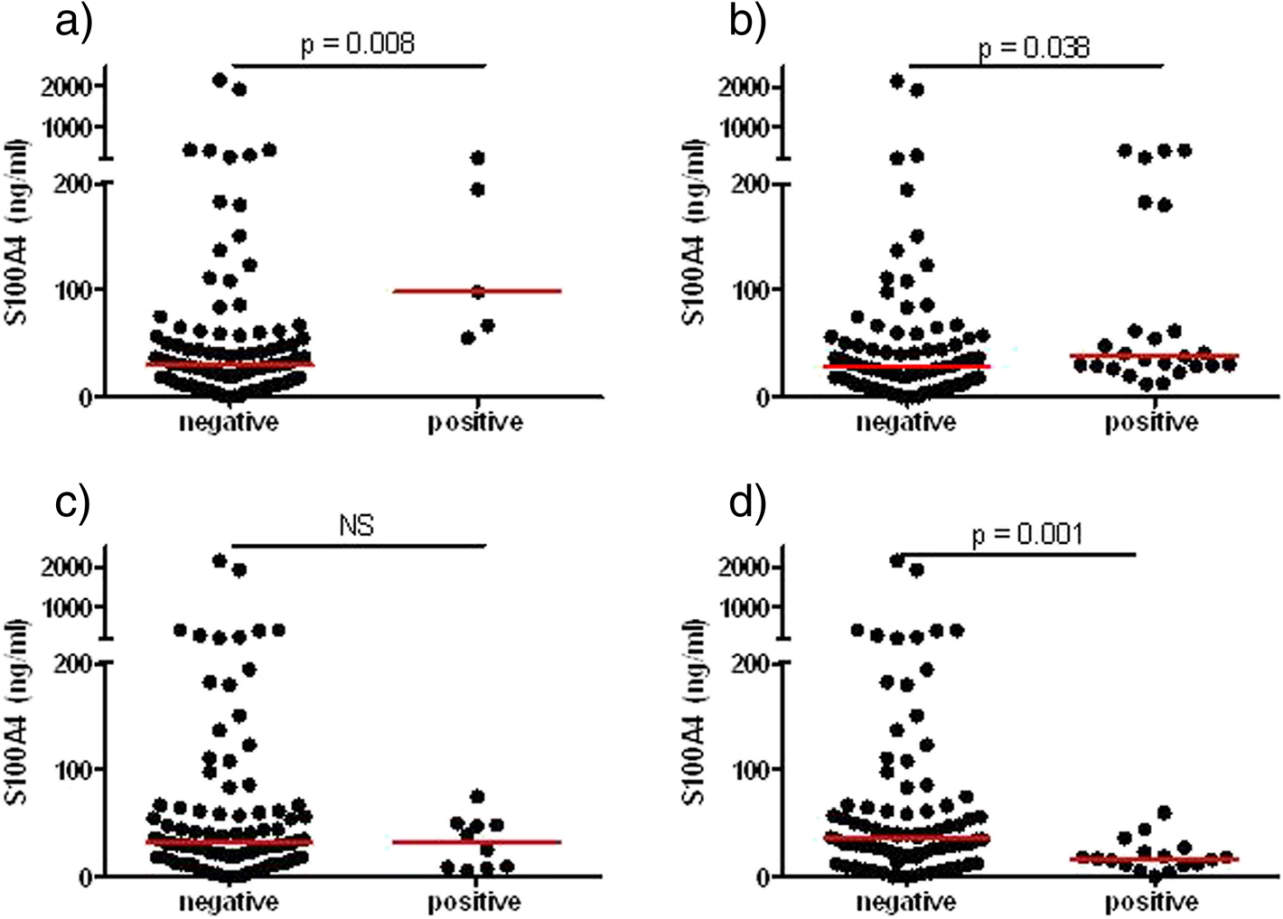
**S100A4 levels in myositis patients divided into two groups based on positive (+) or negative (−) myositis-specific and associated autoantibodies. (a)** Anti-PM-Scl; **(b)** anti-Jo-1; **(c)** anti-Mi-2, **(d)** anti-TIF1 antibodies. Horizontal lines represent the median. The Mann-Whitney *U*-test was used to calculate *P*-values for differences between groups.

Serum levels of S100A4 were compared between patients positive or negative for the myositis-specific and associated autoantibodies directed against Jo-1, Mi-2, PM-Scl and TIF1 antigens (Figure 2). Other autoantibodies were rare and did not allow statistical comparisons. Anti-PM-Scl-positive patients (n = 5) had significantly higher levels of S100A4 compared to anti-PM-Scl-negative patients (n = 99) (98.1 (60.7 to 206.3) versus 30.3 (16.4 to 56.3) ng/ml, *P* = 0.008), and similarly, albeit with a smaller difference, anti-Jo-1-positive patients (n = 25) had higher S100A4 levels than anti-Jo-1-negative patients (n = 79) (38.0 (29.1 to 120.6) versus 28.5 (12.1 to 57.2) ng/ml, *P* = 0.038). No difference was observed between anti-Mi-2-positive (n = 10) and -negative (n = 94) patients (32.4 (8.5 to 48.9) versus 31.5 (18.4 to 61.3) ng/ml, p = 0.440). Surprisingly, anti-TIF1-positive myositis patients (n = 17), who are more likely to develop cancer, had lower levels of S100A4 than anti-TIF1-negative ones (n = 87) (17.1 (11.3 to 25.1) versus 36.6 (21.8 to 66.7) ng/ml, *P* = 0.001).

### Association between S100A4 levels and myositis disease activity

**Figure 3:**
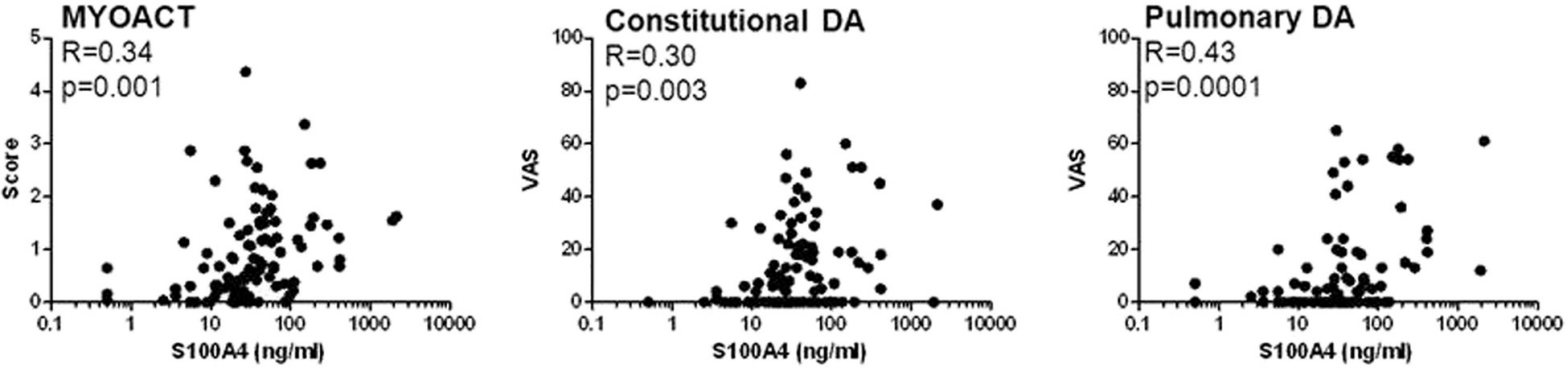
**Correlations of circulating S100A4 levels with myositis disease activity (MYOACT) assessment and its components, constitutional and pulmonary disease activity.** DA, disease activity; VAS, visual analogue scale.

Increased serum levels of S100A4 were associated with several measures of disease activity in myositis patients overall (Figure 3). Specifically, we found a moderate correlation of S100A4 serum levels with the MYOACT score (*r* = 0.34; *P* = 0.001), constitutional (*r* = 0.30; *P* = 0.003), extramuscular (r = 0.36; *P* = 0.0001) and pulmonary disease activity (*r* = 0.43; *P* = 0.0001), but not with skeletal disease activity (*r* = 0.13; *P* =0.208). In addition, there was a weak correlation of S100A4 with CRP (*r* = 0.24; *P* = 0.038) as well as with CK (*r* = 0.27; *P* = 0.015) and moderate correlation with LD (r = 0.37; *P* = 0.002) serum levels. On the other side, there was no difference in S100A4 levels between myositis patients with (n = 32) and without (n = 72) arthritis (32.3 (17 to 65) versus 31.5 (17 to 58) ng/ml, *P* = 0.890).

In patients with PM, S100A4 serum levels moderately correlated with MYOACT (*r* = 0.43; *P* = 0.010) and with constitutional (*r* = 0.43; *P* = 0.011), pulmonary (*r* = 0.44; *P* = 0.008), and extramuscular disease activity (*r* = 0.50; *P* = 0.003). Additional multiple correlations are given online in Additional file 1: Table S1. There was no correlation of S100A4 serum levels with the degree of endomysial inflammatory infiltrate and/or MHC-I overexpression in the muscle biopsies from patients with PM (data not shown). No association was demonstrated between S100A4 and CK (*r* = 0.33; *P* = 0.064), LD (*r* = 0.36; *P* = 0.061), and CRP (*r* = 0.26; *P* = 0.169) serum levels in PM patients.

In patients with DM, S100A4 serum levels correlated moderately with MYOACT (*r* = 0.46; *P* = 0.004) and pulmonary disease activity (*r* = 0.41; *P* = 0.011). Significant correlations were found between S100A4 and CRP levels (*r* = 0.38; *P* = 0.041), but not CK (*r* = 0.09; *P* = 0.631) or LD (*r* = 0.19; *P* = 0.365) serum levels. In addition, we have not observed any significant correlations between S100A4 serum levels and myositis disease activity or laboratory markers in patients with cancer-associated myositis (data not shown).

Multiple regression analysis revealed significant association between S100A4 serum levels, pulmonary disease activity (β = 0.369; *P* = 0.002), LD (β = 0.345; *P* = 0.005) and severity of dysphagia (β = −0.250; *P* = 0.033) in the whole myositis patient group. When analysing the group of PM patients, S100A4 levels were associated only with extramuscular global assessment (β = 0.552; *P* = 0.002), while in the group of DM patients, S100A4 levels correlated with MYOACT (β = 0.557; *P* = 0.003) and CRP (β = 0.391; *P* = 0.029). The group of patients with cancer-associated myositis was too small to perform multiple linear regression analysis. Furthermore, there were no significant bivariate correlations within this group.

## Discussion

In this study we demonstrated 1) no relationship between S100A4 and cancer-associated myositis; 2) increased S100A4 serum levels in myositis patients; 3) a relationship between S100A4 and some myositis-specific and myositis-associated autoantibodies; and 4) association between S100A4 levels and several features of myositis disease activity, particularly with extramuscular symptoms.

There is a body of evidence that S100A4 protein is associated with the development of several cancers and particularly with their ability to metastasize [11,12,14]. Given the association of myositis, and dermatomyositis in particular, with increased risk of cancer development [6,7], it may be surprising that we have not observed higher serum levels of S100A4 in patients with cancer-associated myositis and that S100A4 levels were even lower in those with anti-TIF1 autoantibodies, a biomarker that identifies a large proportion of myositis patients with concomitant cancer [30]. We can speculate that either there was S100A4 consumption in cancer tissue or inability to detect enhanced formation of S100A4 multimeric forms that occur during malignancy [18,31].

Although S100A4 protein was initially studied in malignancy [10], there are several reports demonstrating increased amounts of S100A4 in patients with various inflammatory and autoimmune diseases [17,19-21,32]. In addition, we have previously demonstrated that S100A4 is expressed mainly by infiltrating mononuclear cells, few regenerating muscle fibres and endothelial cells in myositis [22]. In this study, we found increased S100A4 serum levels in all myositis patients and particularly in PM patients compared to those with DM and healthy individuals. Thus, we suggest that S100A4 may either reflect diverse pathological processes that occur in different myositis subtypes and/or that it plays a more important role in PM than in DM.

Furthermore, there was a relationship between circulating S100A4 and the presence of several autoantibodies. We found that the levels of S1004 are elevated in myositis patients positive for PM-Scl and anti-Jo-1, but not Mi-2 antibodies. Both arthralgia and non-erosive arthritis are common in myositis patients, especially in patients with anti-synthetase autoantibodies such as anti-Jo-1 [33]. As disease activity in patients with RA correlated with S100A4 levels [18,23] and anti-Jo-1-positive patients had increased S100A4 serum levels, we expected that presence of arthritis would contribute to the elevation of S100A4 in myositis patients. However, S100A4 levels were comparable between myositis patients with and without arthritis, which may point to a different pathogenesis of joint involvement in myositis patients compared to those with RA. Thus, it can be hypothesised that in myositis, S100A4 may be associated with systemic activation of the immune system rather than with mechanisms driving arthritis during this debilitating disease.

In our present study, we demonstrated weak to moderate correlation between S100A4 serum levels and various myositis disease-activity measures; on the other side, we found no association between serum S100A4 protein levels and muscle strength or changes in the muscle biopsy. These results may be consistent with the findings showing that muscle fibres do not significantly contribute to S100A4 production and myocytes do not respond to stimulation with S100A4 protein by production of pro-inflammatory cytokines [22,34]. Thus, we can speculate that circulating S100A4 protein may reflect the global disease activity, including extramuscular organ involvement, rather than functional muscle impairment in inflammatory myopathy. In this context, association between S100A4 levels and pulmonary disease activity may be of clinical significance. However, these results should be confirmed in further targeted studies.

## Conclusion

In summary, we have demonstrated that circulating S100A4 levels are elevated in myositis patients, especially in those with polymyositis, and correlate with several features of myositis disease activity, particularly with extramuscular involvement, but not with the presence of cancer. Further studies evaluating the role of S100A4 in myositis are needed.

## Additional file

Additional file 1
**Multiple correlations.**

## Abbreviations

aAbs: autoantibodies
AZA: azathioprine
CAM: cancer-associated myositis
CK: creatine phosphokinase
CRP: C-reactive protein
CyA: cyclosporine A
DA: disease activity
DM: dermatomyositis
ELISA: enzyme-linked immunosorbent assay
EMG: electromyography
EQUIV: equivalent
HAQ: health assessment questionnaire
IMACS: International Myositis Assessment & Clinical Studies Group
IQR: interquartile range
LD: lactate dehydrogenase
MMT: manual muscle testing
MTX: methotrexate
MYOACT: Myositis disease activity assessment
NA: not applicable
PM: polymyositis
RA: rheumatoid arthritis
SAS: sulfasalazine
VAS: visual analogue scales
