# Supplementary material for: The metastasis promoting protein S100A4 levels associate with disease activity rather than cancer development in patients with idiopathic inflammatory myopathies
Source: Arthritis Res Ther. 2014 Oct 31;16(5):468. doi: 10.1186/s13075-014-0468-2 (PMC4241220; doi:10.1186/s13075-014-0468-2)
Supplement: Additional file 1 — Multiple correlations. [file 13075_2014_468_MOESM1_ESM.docx]

| **Total sample:** Multiple correlation R=0.563; R2=0.317; Adjusted R2=0.282 | | |  |
| --- | --- | --- | --- |
| **Dependent variable: ln_S100A4ngml** | **Independent variables** | **Standardized coefficients** | **P-value** |
| Variables in the model | ln_Pulmonary Disease Activity | 0,369 | 0.002** |
|  | ln_LD ukatl | 0,345 | 0.005** |
|  | ln_Severity of Dysphagia | -0,25 | 0.033* |
| Excluded variables | sex (0=F, 1=M) | 0,006 | 0,962 |
|  | age | 0,015 | 0,896 |
|  | ln_BMI | -0,147 | 0,182 |
|  | ln_Disease duration | -0,217 | 0,066 |
|  | ln_CK ukatl | 0,182 | 0,325 |
|  | ln_CRP mgl | -0,028 | 0,818 |
|  | ln_Constitutional Disease Activity | 0,162 | 0,178 |
|  | ln_Cutaneous Disease Activity | 0,208 | 0,062 |
|  | ln_Skeletal Disease Activity | 0,014 | 0,902 |
|  | ln_Extramuscular Global Assessment | 0,257 | 0,054 |
|  | ln_Cardiovascular Disease Activity | -0,13 | 0,263 |
|  | ln_Muscle Disease Activity | 0,101 | 0,475 |
|  | ln_MYOACT | 0,259 | 0,09 |
|  | ln_Physician Global Disease Assessment | 0,165 | 0,218 |
|  | ln_HAQ | -0,004 | 0,971 |
|  | ln_reverse_MMT8 | -0,05 | 0,672 |
|  |  |  |  |
| **PM group:** Multiple correlation R=0.552; R2=0.305; Adjusted R2=0.278 | |  |  |
| **Dependent variable: ln_S100A4ngml** | **Independent variables** | **Standardized coefficients** | **P-value** |
| Variable in the model | ln_Extramuscular Global Assessment | 0,552 | 0.002** |
| Excluded variables | sex (0=F, 1=M) | 0,023 | 0,889 |
|  | age | 0,066 | 0,696 |
|  | ln_BMI | -0,08 | 0,664 |
|  | ln_Disease duration | -0,08 | 0,64 |
|  | ln_CK ukatl | 0,213 | 0,22 |
|  | ln_CRP mgl | 0,004 | 0,985 |
|  | ln_LD ukatl | 0,116 | 0,579 |
|  | ln_Constitutional Disease Activity | 0,279 | 0,131 |
|  | ln_Cutaneous Disease Activity | 0,16 | 0,353 |
|  | ln_Skeletal Disease Activity | -0,216 | 0,239 |
|  | ln_Pulmonary Disease Activity | 0,168 | 0,524 |
|  | ln_Gastrointestinal Disease Activity | -0,226 | 0,173 |
|  | ln_Cardiovascular Disease Activity | -0,121 | 0,478 |
|  | ln_Muscle Disease Activity | -0,006 | 0,974 |
|  | ln_MYOACT | 0,086 | 0,783 |
|  | ln_Physician Global Disease Assessment | 0,001 | 0,994 |
|  | ln_HAQ | -0,046 | 0,785 |
|  | ln_reverse_MMT8 | -0,063 | 0,712 |
|  |  |  |  |
| **DM group:** Multiple correlation R=0.650; R2=0.423; Adjusted R2=0.368 | |  |  |
| **Dependent variable: ln_S100A4ngml** | **Independent variables** | **Standardized coefficients** | **P-value** |
| Variables in the model | ln_MYOACT | 0,557 | 0.003** |
|  | ln_CRP mgl | 0,391 | 0.029* |
| Excluded variables | sex (0=F, 1=M) | 0,063 | 0,719 |
|  | age | -0,041 | 0,827 |
|  | ln_BMI | -0,031 | 0,882 |
|  | ln_Disease duration | -0,201 | 0,307 |
|  | ln_CK ukatl | 0,119 | 0,504 |
|  | ln_LD ukatl | 0,021 | 0,915 |
|  | ln_Constitutional Disease Activity | -0,062 | 0,822 |
|  | ln_Cutaneous Disease Activity | 0,107 | 0,712 |
|  | ln_Skeletal Disease Activity | -0,028 | 0,881 |
|  | ln_Pulmonary Disease Activity | 0,135 | 0,506 |
|  | ln_Gastrointestinal Disease Activity | -0,21 | 0,281 |
|  | ln_Cardiovascular Disease Activity | 0,068 | 0,705 |
|  | ln_Muscle Disease Activity | -0,286 | 0,226 |
|  | ln_Physician Global Disease Assessment | -0,311 | 0,273 |
|  | ln_HAQ | -0,252 | 0,217 |
|  | ln_reverse_MMT8 | -0,226 | 0,281 |
|  | ln_Extramuscular Global Assessment | -0,009 | 0,978 |
|  |  |  |  |
